# A facile fabrication method of sericin/chitosan film without additives for fruit coating†

**DOI:** 10.1039/d5ra01962a

**Published:** 2025-06-11

**Authors:** Thi-Hong-No Nguyen, Van-Khanh-Duy Nguyen, Minh-Vuong Phan, Quynh-Nhu Pham, Manh-Huy Do, Thanh-Quang Le, Minh-Ty Nguyen, Thanh-Danh Nguyen

**Affiliations:** a Institute of Advanced Technology, Vietnam Academy of Science and Technology 01A TL29, District 12 Ho Chi Minh city Vietnam danh5463bd@yahoo.com; b Graduate University of Science and Technology, Vietnam Academy of Science and Technology 18 Hoang Quoc Viet, Cau Giay District Hanoi Vietnam

## Abstract

Sericin (SS) serves as a natural adhesive, binding silk fibers within the silkworm cocoon and shielding them from environmental stresses. Commonly, SS-based films rely on additives to improve their physical properties. In this study, we developed an additive-free film composed of SS and chitosan (CS) in an ethanol environment, achieving enhanced tensile strength, elongation at break, and water retention and release capacity through structural modification of SS. The physicochemical properties of the film were comprehensively characterized using FTIR, SEM, and contact angle analyses. Additionally, its antioxidant, antibacterial, and hemocompatibility properties were systematically evaluated, demonstrating its potential for diverse biomedical and environmental applications. Results demonstrated that a film with a SS-to-CS ratio of 2 : 1 exhibited strong DPPH and hydrogen peroxide scavenging activities, induced only 2.07% hemolysis, and displayed moderate antibacterial effects against both Gram-negative (*Escherichia coli*, *Pseudomonas aeruginosa*) and Gram-positive (*Staphylococcus aureus*, *Bacillus subtilis*) bacteria. Biocompatibility and safe use of the SS/CS film were tested on rabbit and human red blood cells. Furthermore, the film was applied for postharvest preservation of fruits, extending the shelf life of bananas up to 8 days and maintaining the freshness of tomatoes for up to 40 days compared to 12 days for untreated samples. These findings highlight the potential of SS/CS films for sustainable agricultural product preservation.

## Abstract 2

In this study, we developed an additive-free film composed of SS and chitosan (CS) in ethanol environment, achieving enhanced tensile strength, elongation at break, and water retention and release capacity through structural modification of SS.

## Graphical abstract

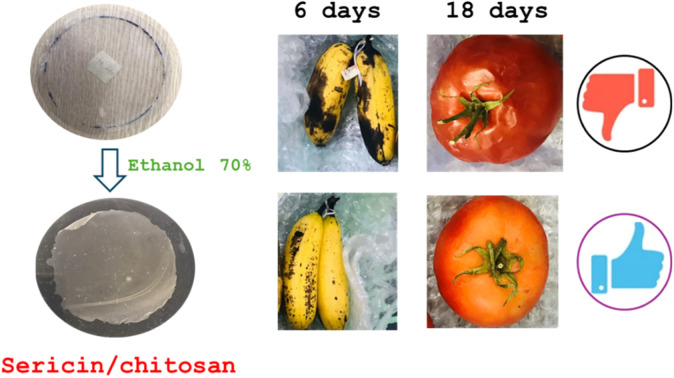

## 1. Introduction

Silk, derived from silkworm cocoons, consists primarily of two proteins: fibroin (FB, 70–80%) and sericin (SS, 20–30%). SS acts as a natural adhesive, binding FB strands to form the robust and durable structure of silkworm cocoons. While FB is extensively utilized in textiles, industrial applications, and medical fields, SS has historically been discarded as a byproduct during silk processing.^1^

With the rapid growth of the silk industry, environmental concerns have intensified due to the large volumes of wastewater produced during silk fiber processing.^2–4^ A major contributor to this issue is the degumming stage, where SS is removed and typically discarded during washing. This process results in the loss of SS and contributes significantly to wastewater pollution.^5,6^ Globally, more than 400 000 tonnes of dried cocoons are processed annually, leading to the release of approximately 50 000 tonnes of dissolved SS into wastewater streams. This presents a pressing environmental challenge, but also a valuable opportunity by developing sustainable methods for SS recovery or reuse, the silk industry could both mitigate its environmental footprint and reclaim a high-value biomaterial.^7,8^

SS is a highly hydrophilic natural polymer with a molecular weight ranging from 20 to 400 kDa, comprising 18 distinct amino acids, several of which are biologically significant. SS exhibits a wide array of biological activities, including enhancing the intestinal absorption of dietary minerals, inhibiting ACE-I activity, lowering blood sugar levels, and demonstrating antioxidant and antibacterial properties.^9,10^ As the demand for biocompatible and biodegradable materials continues to rise, the strategic and scientific utilization of SS holds great potential for the development of bio-products that are both cost-effective and environmentally sustainable.

Chitosan (CS) is widely recognized for biomedical applications and films. With attributes such as biodegradability into harmless polymers, biocompatibility, non-toxicity, and strong adhesive properties, CS has become a pivotal material across various critical fields. Furthermore, the amine groups in CS can be modified or functionalized through intramolecular and intermolecular hydrogen bonding.^11^ Consequently, films derived from SS and CS present a highly promising source of innovative biomaterials.

SS or polysaccharide-based films, whether used individually or in combination, often exhibit brittleness and poor mechanical strength under dry conditions.^12,13^ To address these limitations, previous studies have explored the incorporation of plasticizers such as polyvinyl alcohol (PVA) and glycerol, or reinforcement with metal oxides like MgO and ZnO, to enhance film flexibility and mechanical performance.^14–17^ However, the addition of such modifiers can increase production complexity and cost, while also raising concerns about potential toxicity, particularly in applications involving food packaging, where material safety is paramount. Although PVA and glycerol are generally recognized as safe, residual impurities may still pose health risks. Furthermore, while metal oxides and silver nanoparticles have been shown to improve film properties, their potential cytotoxicity necessitates careful toxicological assessment before widespread use.

To address the aforementioned challenges, this study proposes a simple, additive-free approach for fabricating sericin/chitosan (SS/CS) composite films. By blending sericin with chitosan and applying an ethanol treatment, we induce a conformational transformation in the sericin component. Specifically, ethanol exposure facilitates the transition from a disordered α-helical structure to a more stable and ordered β-sheet configuration. This structural rearrangement, characterized by dense hydrogen bonding within β-sheets, plays a crucial role in enhancing the mechanical strength of the resulting films.^18^ Importantly, our method circumvents the need for synthetic plasticizers or reinforcing agents, thereby reducing potential toxicity and production complexity. As a demonstration of practical utility, the resulting SS/CS films were employed as edible coatings for the preservation of perishable fruits such as tomatoes and bananas.

## 2. Materials and methods

### 2.1. Materials

*Bombyx mori* silk cocoons were gathered in Bao Loc, Vietnam. Chitosan with a molecular weight range from 100 000 to 300 000 Da was purchased from Thermo Scientific (349051000). Triton X100 and DPPH (99.55%) was provided by Sigma-Aldrich. Hydrochloric acid (37%), sodium hydroxide, sodium chloride, potassium chloride, sodium carbonate anhydrous, potassium dihydrogen orthophosphate, disodium hydrogen orthophosphate anhydrous, acetic acid glacial (99.5%) and ethanol (99%) were purchased from Fisher. Rabbit blood was provided by Biotech center of Ho Chi Minh City, Vietnam. Human blood types were gifted by Thi-Hong-No Nguyen and Van-Khanh-Duy Nguyen.

### 2.2. Methods

#### 2.2.1. Sericin extraction

The extraction of silk sericin was performed using a previous method with slight modification.^19^ In brief, 50 grams of silk cocoons was initially chopped into small pieces, followed by the addition of deionized water at a 10 : 1 (w/w) ratio and the mixture was autoclaved at 125 °C for 120 minutes. Following autoclaving, the resulting mixture of SS and FB was filtered to separate the silk FB. After filtration, the SS solution was freeze-dried to obtain the fine powder, put into an amber bottle and kept at cold temperature (∼8 °C).

#### 2.2.2. Preparation of film

Lyophilized SS powder (0.35 g, 1.00 g, or 1.50 g) was thoroughly dissolved in water (25 mL) and constantly agitated for 20 minutes at 50 °C. The CS powder (0.5 g) was dissolved in a 0.1% acetic acid solution (25 mL). At higher concentrations of acetic acid, such as 1.0%, the resulting mixture exhibited reduced homogeneity when blended with the SS solution, indicating that excessive acidity may disrupt the uniform dispersion of components. Until the solution was entirely dissolved, stir continually and kept at 50 °C. A film solution was prepared by slowly adding SS and CS solution at various ratios of 0.7, 2.0, and 3.0. Then, 15 mL solution was transferred to a plastic Petri diameter (11 mm) and allowed to dry at 70 °C for 3 hours. Following this, it was soaked in ethanol for 30 minutes, then dried again at 50 °C for 3 hours before carefully removing the film from the Petri dish. Finally, the film was placed in a desiccator for future studies.

#### 2.2.3. Physicochemical characterizations

##### 2.2.3.1 Water retention and release ability

The water retention and release capacity was evaluated based on a previous study with minor modifications.^20^ Film samples were cut into small pieces measuring 4 × 4 cm. Distilled water (25 mL) was poured to half the volume of a beaker, and the film piece was placed on the rim of the beaker, which was then sealed inside a desiccator. The change in the film mass was recorded every 12 hours. Water retention was determined based on eqn (1).1
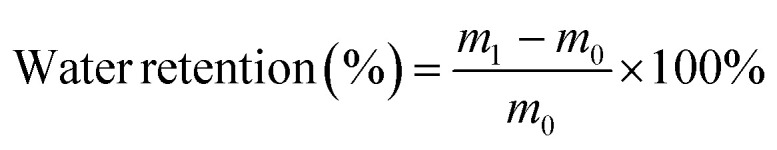
where: *m*_0_ (g) represents the mass of the initial film and *m*_1_ (g) is the film mass after 12 hours.

To study water release, the hydrated film was removed from the desiccator and transferred onto a Petri dish once its mass stabilized (indicating complete hydration). The Petri dish was kept at 25–30 °C, and the film's mass was measured every 12 hours. The water release was calculated using eqn (2).2
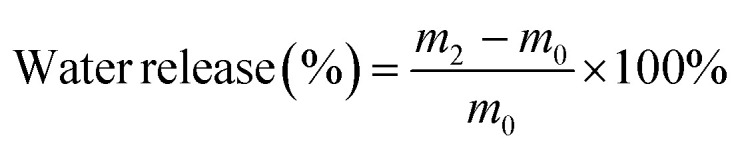
where *m*_0_ (g) represents the mass of the fully hydrated film and *m*_2_ (g) is the film mass after 12 hours on a Petri dish during environmental testing.

##### 2.2.3.2 Mechanical properties

The mechanical properties of the film were evaluated using a previous method.^21^ Tensile strength and elongation at break of the SS/CS film were measured at 25 °C with a Multitest 5-xt apparatus (Mecmesin, UK). Film strips (30 mm × 80 mm) were prepared for testing. A crosshead speed of 100 mm min^−1^ was applied during the measurements. Tensile strength (TS, MPa) and elongation at break (EB, %) were calculated using eqn (3) and (4), respectively.3
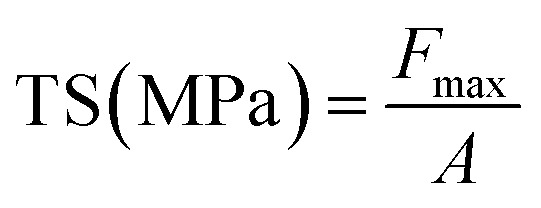
4
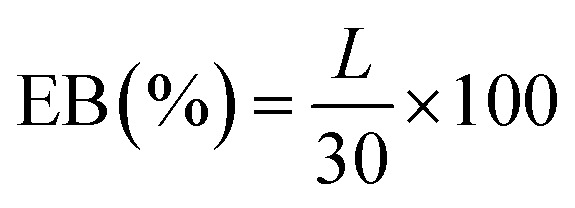
where *F*_max_ is the maximum load (N) applied to rupture the film; *A* is cross-sectional area of the film (mm^2^); *L* is the elongation at rupture (mm).

##### 2.2.3.3 Contact angles and fourier transform infrared spectrophotometer (FTIR)

The surface hydrophobicity of each film was determined using a MicroView USB Digital Microscope, equipped with Analysis Software (220 × 2.0 MP) for image processing. A 1 μL of distilled water was carefully placed on the film surface, and the contact angle (*θ*) at the liquid–solid interface was measured. The average of three measurements, along with the corresponding statistical standard deviation, was reported to ensure accuracy. To evaluate the functional groups present in the films, samples were analyzed using a Bruker FTIR spectrophotometer (Japan) over a scanning range of 400–4000 cm^−1^.

#### 2.2.4. Antioxidant activity

##### 2.2.4.1 DPPH method

Antioxidant activity was evaluated using the 2,2-diphenyl-1-picrylhydrazyl (DPPH) assay, a method based on the reduction of the stable DPPH free radical, which exhibits a characteristic absorption peak at 517 nm.^22^ Briefly, a rectangular film sample (1.0 cm^2^) was submerged in 5.0 mL of ethanol containing 0.05 mM DPPH. The mixture was gently stirred for 30 minutes at room temperature in the absence of light to prevent degradation. Absorbance at 517 nm was measured using a spectrophotometer. A control experiment was conducted under the same conditions, using ethanol in place of the film. The DPPH radical scavenging activity was calculated using eqn (5).5
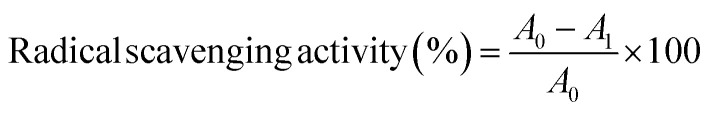
where *A*_0_ and *A*_1_ are the absorbance of the control and sample, respectively.

##### 2.2.4.2 Hydrogen peroxide scavenging assay

The hydrogen peroxide scavenging assay was performed as described by a previous method with minor modifications.^23^ Briefly, 0.25 mL of ferrous ammonium sulfate (1 mM) was mixed with 1.5 mL of the sample. Next, 62.5 μL of hydrogen peroxide (5 mM) was added, and the mixture was incubated at room temperature in the dark for 5 minutes to prevent hydrogen peroxide photobleaching. After incubation, 1.5 mL of 1,10-phenanthroline (1 mM) was added to the mixture, which was thoroughly mixed and left to stand at room temperature for 10 minutes. The absorbance was measured at 510 nm using a spectrophotometer. A blank solution, comprising 1.5 mL of 1,10-phenanthroline (1 mM), 1.562 mL of water, and 0.25 mL of ferrous ammonium sulfate (1 mM), was prepared, and its highest absorbance value was used as a reference. The hydrogen peroxide scavenging capacity of the films was calculated using eqn (6).6
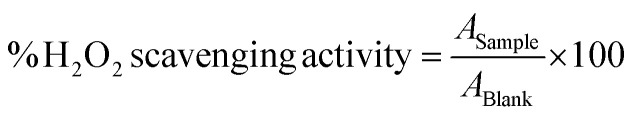


#### 2.2.5. Antibacterial abilities

To evaluate antibacterial activity, an agar medium was prepared containing 10 g per L peptone, 5 g per L meat extract, 10 g per L NaCl, and 10 g per L agar, following the method described previously.^24^ The medium was used to activate the test bacteria including two negative Gram strains (*Escherichia coli* and *Pseudomonas aeruginosa*) and two positive Gram strains (*Staphylococcus aureus* and *Bacillus subtilis*) at room temperature for 24 hours. Petri dishes with diameter of 90 mm were then prepared with meat–peptone agar, and the test bacteria were reactivated and suspended in a suitable medium, then diluted to reach a concentration of 10^8^ CFU mL^−1^, corresponding to a 0.5 McFarland standard at a wavelength of 600 nm. For the inoculation, 1 mL of this culture was uniformly spread over the surface of the agar plate. A 2 mm diameter well was created in the agar using a sterile punch, and 10 μL of each film solution was carefully added to the well. Distilled water served as the negative control, while ampicillin (300 ppm) was used as the positive control. The Petri dishes were incubated at 37 °C for 24 hours, after which the antibacterial activity of the film was assessed by observing the formation of inhibition zones around the wells.

#### 2.2.6. Hemolysis testing

Red blood cells (RBCs) were isolated from 2 mL of anticoagulated rabbit blood treated with EDTA. The blood sample was centrifuged at 3000 rpm for 20 minutes to separate the RBCs, which were subsequently washed twice with phosphate-buffered saline (PBS) to remove residual plasma. A film sample was then carefully added to the RBC suspension. Triton X-100 and PBS were used as positive and negative controls, respectively. The mixture was incubated at 37 °C for 1 hour, followed by centrifugation at 3000 rpm for 15 minutes. The absorbance of the resulting supernatant was measured at 540 nm to assess hemolysis, following the method described by a previous report.^25^ The percentage of hemolysis was calculated to evaluate the film's effect on RBC integrity by eqn (7).7
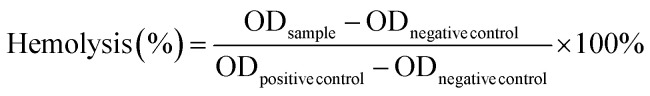


#### 2.2.7. Food coating

Solutions of varying concentrations were prepared for the experiment, utilizing pesticide-free, thoroughly washed, and clearly labeled *Areca bananas* and *Campari tomatoes*. In this study, the film-forming solution was used as an edible coating through the dipping method, which is one of the commonly used techniques for applying edible packaging directly onto the surface of fruits, especially at the post-harvest stage. This method allows for better surface contact and uniform distribution of the active components. Each sample was immersed in the prepared solutions and air-dried on a rack at room temperature to ensure no risk of film contamination. After drying for 24 hours, the samples were gently wrapped in film to simulate the “shipping environment” typically encountered during export, following the method described by ref. 15. The change in sample mass after 24 hours was calculated using eqn (8).8
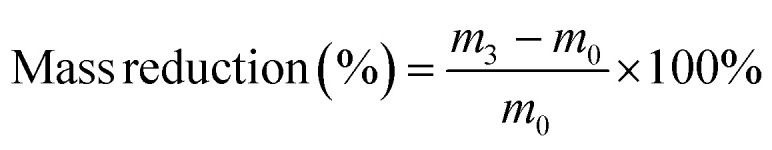
where *m*_0_ and *m*_3_ (g) are the mass of the initial sample and the sample every 24 hours, respectively.

## 3. Result and discussion

### 3.1. Fabrication of film

The sericin-based film has been extensively studied for its mechanical properties, with additives such as PVA and glycerol commonly used to enhance its strength. However, these additives may compromise safety and complicate the production process. To address this, we developed a straightforward method by blending SS and CS, followed by drying under ambient conditions. The fabricated films were subsequently soaked in ethanol to improve their mechanical properties.

**Fig. 1:**
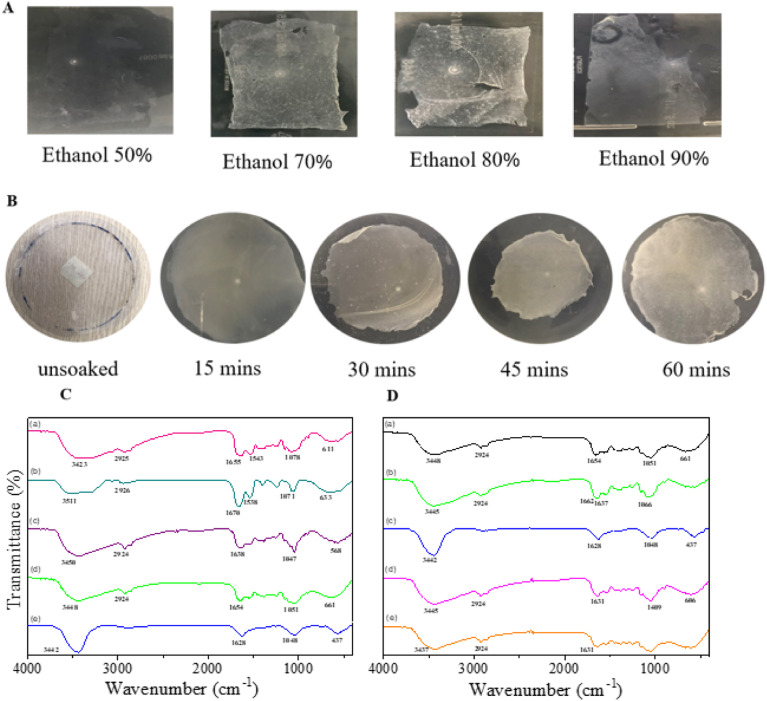
Images of SS-to-CS ratio of 2 : 1 film treated with different concentrations of ethanol (A), and treated with ethanol 70% at different times (B); and FT-IR spectra of different films (C): chitosan (a), SS (b), SS treated with ethanol (c), SS/CS film (d) and SS/CS film treated with ethanol (e); and SS-to-CS ratio of 2 : 1 film treated in ethanol at different time (D): without ethanol (a), 15 min (b), 30 min (c), 45 min (d), and 60 min (e).

To evaluate the effect of ethanol concentration, the films were treated with varying concentrations ranging from 50% to 90%. As shown in Fig. 1A, films treated with 50% or 90% ethanol could not be removed from the Petri dish, while treatment with 80% ethanol induced strong contraction of film, leading to its structural disruption. The optimal ethanol concentration for treating films after the drying process was determined to be 70%. The soaking time was further optimized by treating the films with 70% ethanol for 15, 30, 45, and 60 minutes (Fig. 1B). Results indicated that complete removal of the film from the Petri dish occurred after 30 minutes of soaking. This finding confirms that soaking in 70% ethanol for 30 minutes significantly enhances the membrane formation of SS/CS films.

To analyze structural changes induced by ethanol treatment, FT-IR spectroscopy was conducted on both individual components and films. The FT-IR spectrum of chitosan revealed a strong band at 3423 cm^−1^, corresponding to N–H and O–H stretching and intramolecular hydrogen bonding. Peaks at 2925 and 2850 cm^−1^ were assigned to symmetric and asymmetric C–H stretching, characteristic of polysaccharides. The residual *N*-acetyl groups were identified by a band at 1655 cm^−1^ (C=O stretching of amide I), and a band at 1543 cm^−1^ corresponded to N–H bending of amide II. Additionally, a band at 1078 cm^−1^ was attributed to C–O stretching.

The structural changes in sericin were also examined using FT-IR spectroscopy. Ethanol-treated solid SS and SS/CS samples exhibited significant differences compared to untreated samples. In ethanol-treated samples, peaks at 1638 cm^−1^ and 1626 cm^−1^, corresponding to the β-sheet conformation of sericin molecules (amide I group), were observed. In contrast, untreated samples displayed peaks at 1680 cm^−1^ for SS and 1654 cm^−1^ for SS/CS films, indicative of an α-helix structure.^26^ The SS/CS films also showed characteristic peaks associated with both chitosan and sericin.

To further confirm the effect of soaking time on film structure, FT-IR spectra of samples treated with 70% ethanol for 15, 30, 45, and 60 minutes were measured. Results revealed that the β-sheet conformation, initially observed at 1654 cm^−1^, shifted to 1637 cm^−1^ after 15 minutes of soaking and to 1628 cm^−1^ after 30 minutes, with minimal changes beyond this point. These findings confirm that sericin in the films predominantly adopts a β-sheet conformation after ethanol treatment. Overall, the results demonstrate that ethanol concentration and soaking time play critical roles in improving the mechanical properties and structural integrity of SS/CS films. The optimal conditions established in this study provide a simple yet effective strategy for enhancing film performance while maintaining safety and production efficiency.

**Fig. 2:**
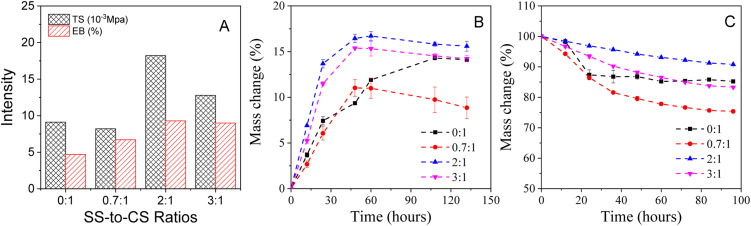
Tensile strength (TS) and elongation at break (EB) intensity (A); water absorption (B) and releasing ability (C) of the films at various SS-to-CS ratios of 0 : 1, 0.7 : 1, 2 : 1, 3 : 1.

The study investigated the fabrication and mechanical properties of films at various sericin-to-chitosan (SS-to-CS) ratios, focusing on tensile strength (TS) and elongation at break (EB), as illustrated in Fig. 2A. The results demonstrated that SS significantly enhanced the mechanical properties of chitosan films. Specifically, the maximum tensile strength increased markedly from 9.11 × 10^−3^ MPa for the pure chitosan film to 18.22 × 10^−3^ MPa at an SS-to-CS ratio of 2 : 1, likely due to the abundance of hydroxyl groups in SS. These groups form hydrogen bonds, enhancing cross-linking between polysaccharides, thereby strengthening the film matrix and improving the mechanical property.^20^ However, this property declined to 12.78 × 10^−3^ MPa at an SS-to-CS ratio of 3 : 1. Similarly, the EB values increased significantly from 4.7% for the pure chitosan film to 9.3% at an SS-to-CS ratio of 2 : 1, before slightly decreasing to 9.0% at a ratio of 3 : 1. This improvement in flexibility may be attributed to increased molecular chain mobility facilitated by sericin penetration.^16^

The water retention and release capacities of films at different SS-to-CS ratios were assessed under sterile conditions, with controlled humidity in a desiccator (Fig. 2B and C). Films with a 2 : 1 SS-to-CS ratio exhibited the highest water absorption, increasing by nearly 17% after 48 hours. This high hygroscopicity suggests that such films are particularly effective at moisture absorption, making them suitable for food protection applications. After 48 hours, water absorption plateaued, indicating stable retention, with the 3 : 1 ratio film maintaining consistency even after 132 hours, unlike other formulations that showed a gradual decline over time. In contrast, films with a 3 : 1 ratio displayed slightly lower hygroscopicity over the same period.

In terms of water release, films with lower sericin content (SS-to-CS ratio of 0.7 : 1) released significantly more water, reaching 18.4% over 48 hours. Conversely, films with a 2 : 1 ratio showed minimal water release (4.4%), indicating superior stability in preserving water content while gradually releasing it into the environment. This performance highlights the potential of films with a 2 : 1 SS-to-CS ratio for further physicochemical characterization and practical applications in food protection.

### 3.2. Physicochemical characterizations

**Fig. 3:**
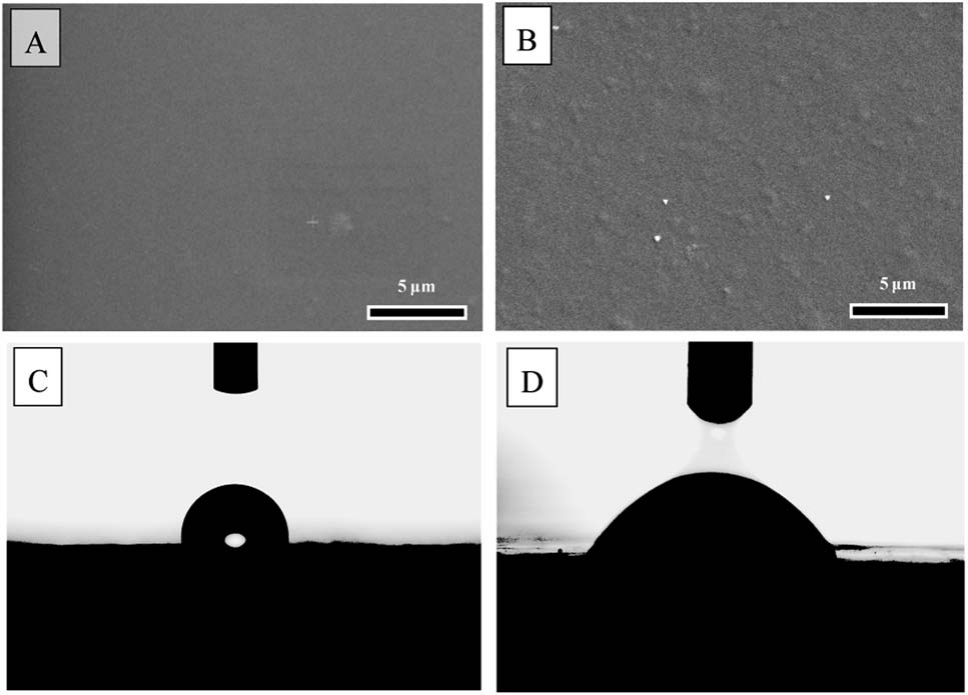
SEM micrographs and contact angle of the film fabricated from chitosan (A and C) and SS-to-CS ratio of 2 : 1 (B and D).

Scanning electron microscopy (SEM) was employed to examine the morphology of CS and SS/CS films (Fig. 3A and B). The results revealed that the CS film exhibited a smoother, flatter, and more uniform surface morphology compared to the SS/CS film. This observation suggests that the incorporation of sericin induces cross-linking with the polysaccharide chains of chitosan, resulting in a rougher surface. This structural modification enhances the mechanical properties and water absorption capacity of the SS/CS film, confirming the successful fabrication of cross-linked films.

The water contact angle measurements for the films are shown in Fig. 3C and D. The contact angles (*θ*) for CS and SS/CS films were determined to be 96.5° and 62.2°, respectively. This indicates that the CS film is hydrophobic, while the SS/CS film is hydrophilic. The increased hydrophilicity of the SS/CS film is attributed to the presence of sericin, which promotes water absorption.

### 3.3. Biological activity

#### 3.3.1. Antioxidant activity

Antioxidant activity plays a dual role in food systems, potentially accelerating spoilage while posing dietary health risks. Incorporating antioxidants into packaging materials can significantly enhance their ability to preserve the nutritional value, quality, and safety of food products.^22^ In this study, the antioxidant properties of SS/CS films were evaluated using DPPH and H_2_O_2_ radical scavenging assays. The films exhibited a DPPH radical scavenging activity of 40.3% and an H_2_O_2_ scavenging activity of 63.9%. These activities are based on the ability to donate or accept electrons, forming O–H or N–H bonds with free radicals. Typically, the tightly bonded internal network of chitosan limits its antioxidant activity.^27^ However, the inclusion of SS in the films significantly enhances their free radical scavenging ability. SS disrupts the strong intramolecular OH and NH_2_ bonds in CS, thereby improving antioxidant performance. This enhancement is attributed to the short-chain peptides in sericin, which can stabilize free radicals by transforming them into less reactive products.^28^

#### 3.3.2. Antibacterial activity

**Fig. 4:**
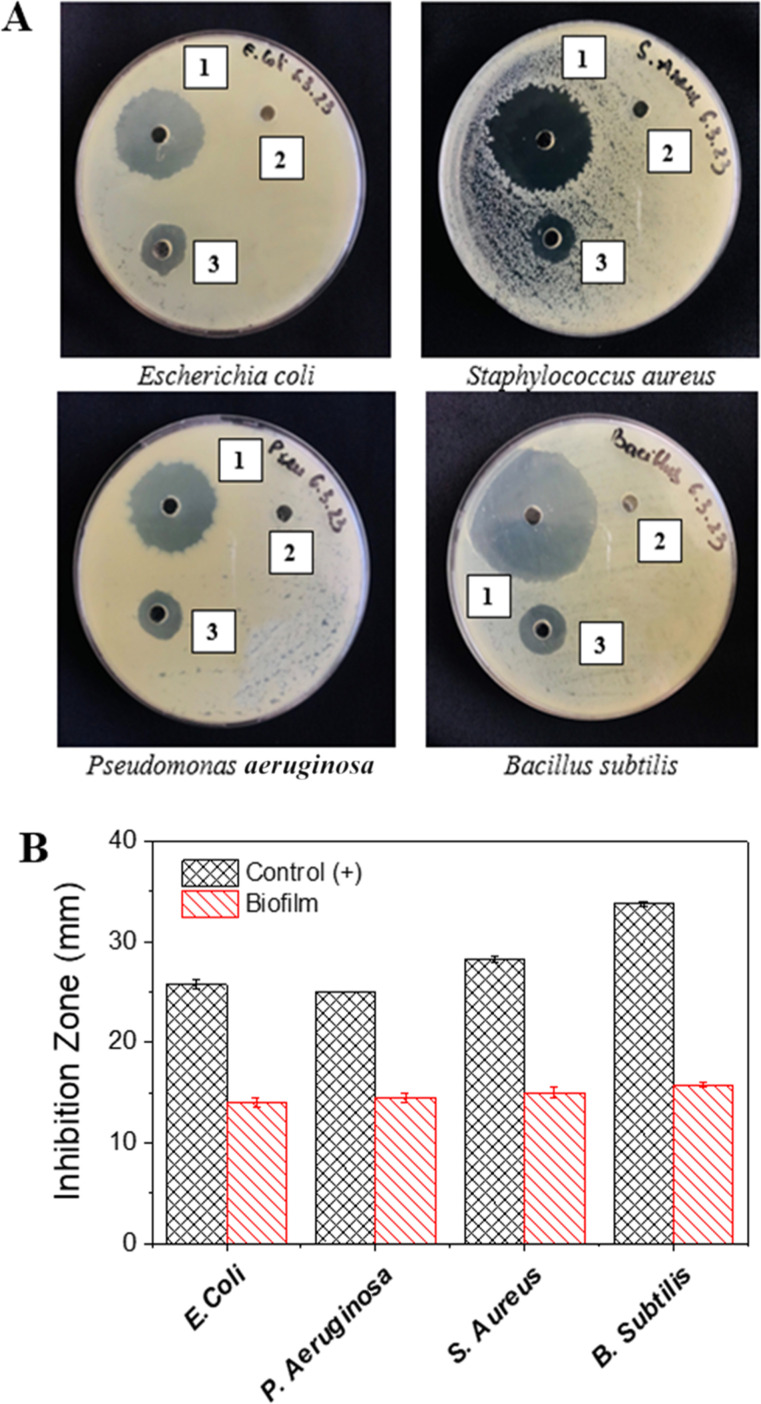
The images (A) of antibacterial activity observed after 24 hours include: (1) ampicillin at 300 ppm (positive control); (2) distilled water (negative control); and (3) the film with SS-to-CS ratio of 2 : 1; and plots of inhibition zones vesus various bacterial strains (B).

The antibacterial activity of films depends on the diffusion of their active components, as evidenced by the formation of inhibition zones around paper discs placed on agar plates. In this study, the antibacterial efficacy of SS/CS film was tested against two Gram-negative strains (*E. coli* and *P. aeruginosa*) and two Gram-positive strains (*S. aureus* and *B. subtilis*) (Fig. 4). The negative control showed no inhibition zones, while the positive control (300 ppm ampicillin) showed strong antibacterial activity, with inhibition zone diameters ranging from 26.50 ± 0.50 mm to 33.75 ± 0.25 mm. Remarkably, the SS/CS films exhibited inhibition zone diameters between 14.00 ± 0.50 mm and 15.75 ± 0.25 mm for all tested bacterial strains, highlighting their potential for applications in packaging and biomedical fields.

The antibacterial activity of SS/CS films was more pronounced against Gram-positive bacteria compared to Gram-negative bacteria, a difference attributed to the structural disparities in their cell walls. Gram-positive bacteria have a thick peptidoglycan layer, which is more susceptible to antibacterial agents, whereas Gram-negative bacteria possess a more complex cell wall structure, including a thin peptidoglycan layer, a periplasmic space, and an outer membrane composed of lipopolysaccharides and lipoproteins. This multilayered architecture enhances resistance of Gram-negative bacteria to antibacterial agents.^29,30^ These findings are consistent with previous studies. For example, sericin/agar films demonstrated inhibition zones of 22.1 ± 0.04 mm for Gram-positive bacteria and 17.4 ± 0.05 mm for Gram-negative bacteria.^16^ Similarly, chitosan/silk sericin 3D porous structures showed inhibition zones of 8.5 ± 0.2 mm for Gram-positive bacteria and 8.0 ± 0.3 mm for Gram-negative bacteria.^31^ This comparison underscores the promising antibacterial performance of SS/CS films.

#### 3.3.3. Hemolysis testing

**Fig. 5:**
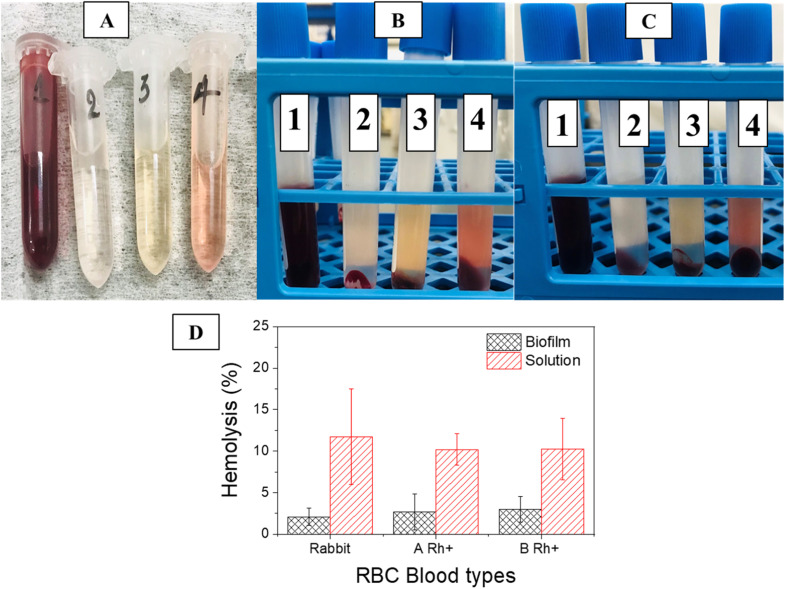
The solution after performing hemolysis testing: rabbit RBCs (A), human RBCs A Rh+ blood (B) and human RBCs B Rh+ blood (C) with (1): Trixton X-100; (2): PBS; (3): SS/CS film; and (4): SS/CS solution; and plots of hemolysis against RBC blood types (D).

Hemolysis testing is a critical method for evaluating the biocompatibility of biomaterials.^32,33^ In this study, the hemolytic activities of both SS/CS solution and SS/CS film were investigated as shown in Fig. 5. The results revealed that the film exhibited significantly lower hemolytic activity compared to the solution, underscoring its superior biocompatibility. Specifically, the SS/CS solution resulted in a hemolysis rate of 11.74%, a level considered harmful to RBCs. This elevated hemolysis rate is attributed to the 0.5% acetic acid used as a solvent for dissolving chitosan, which disrupts RBC membranes.

In contrast, the SS/CS film demonstrated a hemolysis rate of just 2.07%, falling within the safe range for biomedical applications. Consistent results were observed in tests with human and rabbit RBCs, with hemolysis rates of 2.68% and 2.96% for blood types A Rh+ and B Rh+, respectively. These findings confirm excellent biocompatibility of the SS/CS film and its potential for safe use in food and biomedical applications.

### 3.4. Fruit coating

#### 3.4.1. Areca bananas coating

**Fig. 6:**
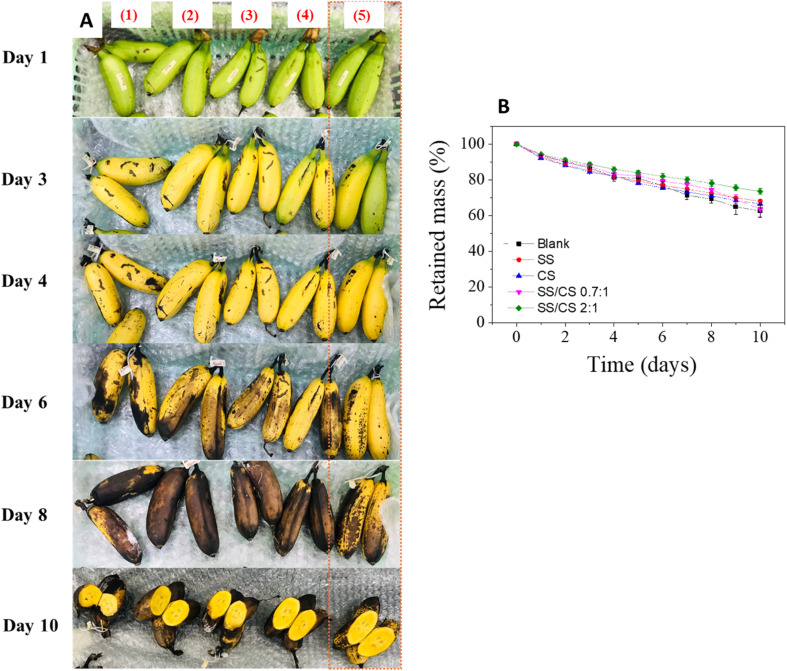
The photos (A) of bananas packaged by films changed over time: (1) – blank, (2) – sericin, (3) – chitosan, (4) – SS/CS (0.7 : 1); (5) – SS/CS (2 : 1); and plots of retained mass *versus* storage time of bananas (B).

The coating of *Areca bananas* was conducted using various films, including SS, CS, and two SS/CS blends with ratios of 0.7 : 1 and 2 : 1. A control sample without film treatment was included for comparison. Bananas were stored at room temperature to mimic natural transport conditions. Changes in sample mass and visual appearances are summarized in Fig. 6.

After 3 days of storage, all banana samples ripened uniformly, with the SS/CS (2 : 1) samples standing out for their ability to retain a green, unripe appearance. By 5 days, while the treated samples exhibited impressive maturation, the control sample developed black spots, a deterioration not observed in film-treated bananas. Notably, the SS/CS (2 : 1) film preserved the green color of the stems and showed no discoloration. After 8 days, the untreated control had completely rotted, with black stains covering more than half of the fruit, whereas the treated samples only began showing slight darkening. Remarkably, the SS/CS (2 : 1) film maintained the highest fruit quality, producing visually appealing, ripe bananas. These findings demonstrate the superior efficacy of the SS/CS (2 : 1) film in extending the shelf life of *Areca bananas* to at least eight days.

Weight loss measurements further highlighted the advantages of the SS/CS (2 : 1) film, which exhibited the least weight loss after 8 days of storage. Specifically, the SS/CS (0.7 : 1) and SS/CS (2 : 1) films preserved 74.4 ± 1.8% and 78.9 ± 1.7% of the initial weight, respectively, compared to 69.2 ± 2.2% in the control sample. This superior performance is attributed to the film's ability to protect the fruit from microbial contamination and reduce its respiration rate, thereby prolonging freshness. Overall, the results suggest that the SS/CS (2 : 1) film is a highly effective solution for preserving *Areca bananas* post-harvest.

#### 3.4.2. *Ansal tomatoes* coating

The preservation procedure for tomatoes followed a methodology similar to that used for *Areca bananas*. Tomato samples were treated with film solutions including SS, CS, SS/CS (0.7 : 1), SS/CS (2 : 1), and SS/CS (3 : 1). Following natural drying, the tomatoes were stored at room temperature under simulated transport conditions.

External observations of *Ansal tomatoes* highlighted notable differences in preservation quality among the treatments. After 7 days, all ripe tomatoes displayed a vibrant red color, with the treated samples appearing particularly appealing. By 12 days, the untreated control samples had softened and darkened more significantly than those treated with films. Among the treated samples, the SS/CS (2 : 1) film stood out, maintaining the tomatoes' attractive round shape and bright red color, while other treatments resulted in drier and rougher skins. After 18 days, the control samples exhibited severe spoilage, becoming mushy, emitting an unpleasant odor, and showing fallen fruit stalks. In contrast, the film-treated samples retained their firmness and dryness, although some wrinkling of the skin was observed. Notably, the SS/CS (2 : 1) film preserved the tomatoes' smooth peel and vibrant color, showing no significant signs of damage even after 40 days of storage.

**Fig. 7:**
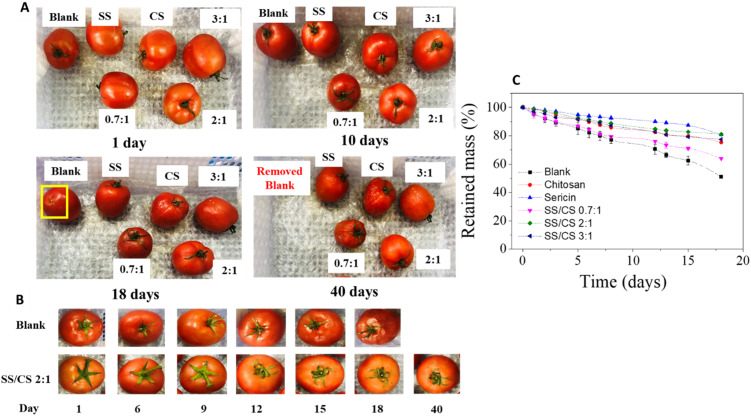
The photos of tomatoes packaged by all films (A) and comparison between blank SS/CS 2 : 1 film samples (B) changed over time and plots of retained mass *versus* storage time of tomatoes (C).

Weight loss analysis revealed that tomatoes treated with films experienced significantly less weight loss compared to the control samples (Fig. 7C). After 18 days, retained weights were as follows: 75% for chitosan-treated tomatoes, 80.7% for sericin-treated tomatoes, 63% for SS/CS (0.7 : 1), 81.0% for SS/CS (2 : 1), and 77.4% for SS/CS (3 : 1). In contrast, the control sample retained only 51% of its weight. These results confirm the superior efficacy of the SS/CS (2 : 1) film, which effectively extended the post-harvest quality and appearance of tomatoes for up to 40 days, far exceeding the 10-day shelf life of untreated samples. Similar results were reported in a previous study utilizing sericin-based edible coatings supplemented with 1.0% *Aloe vera* and 1.5% glycerol, highlighting the potential of such film formulations for fruit preservation.^15^

## 4. Conclusion

This study successfully developed a film incorporating chitosan and sericin as crosslinking agents for polysaccharides treated with ethanol 70%. Sericin in the films after ethanol treatment, have been demonstrated to predominantly adopt a β-sheet conformation. The films showed desirable characteristics, including transparency, visual appeal, flexibility, and ease of separation. Comprehensive analyses revealed its functional properties, such as strong antioxidant activity and effective antibacterial activity against both Gram-negative (*E. coli*, *P. aeruginosa*) and Gram-positive (*S. aureus*, *B. subtilis*) bacterial strains. Biocompatibility and safe use of the SS/CS film were tested on rabbit and human red blood cells with hemolysis rate of just 2.07%. The application of a coating with an SS-to-CS ratio of 2 : 1 on *Areca bananas* extended the storage life to 8 days while maintaining quality and appearance. For *Ansal tomatoes*, this coating preserved the fruits for over 40 days, significantly reducing spoilage and weight loss. These findings underscore the potential of sericin/chitosan films as a promising solution for post-harvest preservation, effectively extending the shelf life of agricultural products and minimizing losses during storage and transportation.

## Author contributions

Thi-Hong-No Nguyen: investigation, formal analysis, validation, software, originate manuscript; Van-Khanh-Duy Nguyen, Minh-Vuong Phan, Quynh-Nhu Pham, Manh-Huy Do, Thanh-Quang Le, Minh-Ty Nguyen: investigation, visualization, validation; Thanh-Danh Nguyen: conceptualization, supervisor, writing – review & editing.

## Conflicts of interest

The authors of this paper state that they have no competing financial interests or personal relationships that could have influenced the reported work.

## Supplementary Material

RA-015-D5RA01962A-s001

## Data Availability

The data of this article, including data of retained mass *versus* storage time of bananas and tomatoes are available in the ESI.†.
