# Supplementary material for: A facile fabrication method of sericin/chitosan film without additives for fruit coating
Source: RSC Adv. 2025 Jun 11;15(25):19704–13. doi: 10.1039/d5ra01962a (PMC12153330; doi:10.1039/d5ra01962a)
Supplement: RA-015-D5RA01962A-s001 [file RA-015-D5RA01962A-s001.pdf]

Supplementary Information (SI) for RSC Advances.
This journal is © The Royal Society of Chemistry 2025

A FACILE FABRICATION METHOD OF SERICIN/CHITOSAN FILM WITHOUT ADDITIVES FOR FRUIT COATING

Thi-Hong-No Nguyen[1,2], Van-Khanh-Duy Nguyen[1], Minh-Vuong Phan[1], Quynh-Nhu Pham[1], Manh-Huy Do[1], Thanh-Quang Le[1], Minh-Ty Nguyen[1], Thanh-Danh Nguyen[1,2]*

[1]Institute of Advanced Technology, Vietnam Academy of Science and Technology, 01A TL29, District 12, Ho Chi Minh city, Vietnam.

[2]Graduate University of Science and Technology, Vietnam Academy of Science and Technology, 18 Hoang Quoc Viet, Cau Giay District, Hanoi, Vietnam.

*Corresponding email: danh5463bd@yahoo.com

Supporting Information

Figure S1. Data of retained mass versus storage time of bananas

Days	Blank		Sericin		CS		SS/CS (0.7:1.0)		SS/CS (2:1)	
	Mean	SD	Mean	SD	Mean	SD	Mean	SD	Mean	SD
0	100	0	100	0	100	0	100	0	100	0
1	93.66	0.14	92.91	0.11	92.29	0.21	93.85	0.53	94.27	0.74
2	89.90	0.10	88.43	0.17	88.06	0.05	90.42	0.76	91.18	1.06
3	86.68	0.05	85.15	0.17	84.41	0.01	87.53	0.99	88.62	1.23
4	81.48	2.34	81.78	0.06	82.01	0.15	83.12	2.34	85.88	1.40
5	80.84	0.15	79.31	0.04	78.14	0.17	82.14	1.27	83.93	1.47
6	76.71	2.11	76.85	0.10	75.50	0.24	79.18	2.14	81.85	1.63
7	71.33	2.37	74.83	0.19	73.25	0.23	77.45	1.60	80.14	1.62
8	69.21	2.24	72.54	0.23	71.03	0.14	74.44	1.83	78.09	1.74
9	64.90	4.43	69.89	0.35	68.39	0.01	69.08	2.60	75.56	1.70
10	62.63	3.67	67.98	0.27	66.51	0.05	63.57	4.25	73.46	1.68

Figure S2. Data of retained mass versus storage time of tomatoes

Days	Blank		Sericin		CS		SS/CS (0.7:1)		SS/CS (1:1)		SS/CS (3:1)	
	Mean	SD	Mean	SD	Mean	SD	Mean	SD	Mean	SD	Mean	SD
0	100	0	100	0	100	0	100	0	100	0	100	0
1	95.50	1.76	98.95	0.12	98.87	0.01	95.12	2.80	98.68	0.26	97.08	0.39
2	92.15	3.12	97.97	0.15	97.36	0.49	92.28	2.08	97.18	0.62	95.54	0.53
3	89.41	2.75	97.11	0.05	94.73	0.21	89.82	1.84	95.98	0.98	92.94	0.03
5	85.12	4.39	94.74	0.04	91.52	0.25	86.75	0.31	92.38	0.69	91.61	0.26
6	82.16	3.49	93.95	0.17	89.68	0.70	84.36	0.20	91.32	0.60	90.50	0.54
7	79.87	3.32	93.28	0.03	87.52	0.57	81.17	1.05	89.83	0.01	88.95	1.34
8	77.03	2.33	92.61	0.01	85.71	0.08	79.38	0.49	88.63	0.47	87.00	0.29
12	70.58	3.55	89.93	0.44	82.50	0.63	75.96	0.30	84.58	0.46	82.78	0.47
13	66.41	1.66	89.11	0.07	80.71	0.30	73.33	2.19	83.70	0.73	80.73	0.11
15	62.43	3.19	87.29	0.90	79.68	0.16	71.07	0.78	82.71	0.04	79.21	0.29
18	51.03	0.02	80.72	0.04	75.31	0.01	63.89	0.03	81.08	0.05	77.40	0.06
